# High-Accuracy Quantitative Nuclear Magnetic Resonance Using Improved Solvent Suppression Schemes

**DOI:** 10.1021/acs.analchem.5c01139

**Published:** 2025-09-25

**Authors:** Bruno C. Garrido, Lucas J. Carvalho, Ian W. Burton, Pearse McCarron

**Affiliations:** † Metrology Research Centre, National Research Council Canada, 1411 Oxford Street, Halifax, Nova Scotia B3H 3Z1, Canada; ‡ Organic Analysis Laboratory, Quality and Technology, 119536 National Institute of Metrology, Avenida Nossa Senhora das Graças, 50, Duque de Caxias 25250-020, RJ, Brazil; § Aquatic and Crop Resources Development, National Research Council Canada, 1411 Oxford Street, Halifax, Nova Scotia B3H 3Z1, Canada

## Abstract

Quantitative nuclear magnetic resonance (qNMR) has contributed to reliable and accurate measurements of organic compounds enabling quantitation even when no standards of the specific compounds are available. Such high-accuracy determinations are critical across the field of analytical chemistry, with the advances in qNMR being of utmost importance in the production of reference standards for a range of organic compounds. The ability to perform these accurate measurements in the presence of natural isotopic abundance solvents is important for increasing throughput and expanding the number of applications that benefit. In this work, we have assessed several pulse sequences for solvent suppression. The limitations of NMR acquisitions in the presence of large solvent signals and solvent suppression such as limited dynamic range, losses due to relaxation and proximity to the solvent peak where quantitation starts failing were studied in depth and discussed. We have shown that binomial-like sequences produce the most robust and reliable results in the majority of scenarios and propose alternative sequences using modern pulses that produce satisfactory results in situations where the most accurate sequences are not applicable. We present the development and use of binomial-like pulses in an inversion–recovery sequence that allows T1 measurement in experiments without deuterated solvent (no-D NMR) to enable the use of correct repetition times for high-accuracy measurements under these conditions. Although the binomial-like sequences present the limitation of having secondary suppression notches, there is enough flexibility to adjust the position of those notches. Finally, we present a full measurement uncertainty budget estimation including all uncertainty allowances that are relevant when solvent suppression is used.

## Graphical abstract

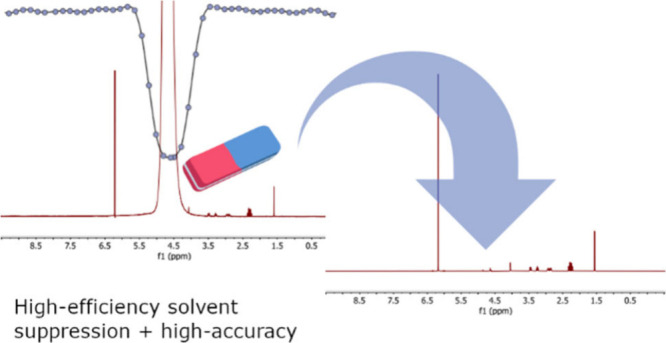

## Introduction

Nuclear magnetic resonance spectroscopy (NMR) is among the most versatile and informative spectroscopic techniques employed in chemical research.^1^ Although its quantitative potential has been known for several decades, it was only in the late 1990s that it was explored as an analytical tool to provide accurate and reliable quantitative results.^2–4^ Its adoption as a primary quantitative technique was transformative in organic analytical chemistry. The vast majority of methods in the field require the use of standards (reference materials) of the same compound for calibration, whereas quantitative nuclear magnetic resonance (qNMR) does not. This capability enables reliable quantitative measurements, even when standards of the same compound do not exist, which, along with the nondestructive nature of NMR, provides a powerful basis for advancing chemical measurements in different fields of study.

Although qNMR is now a method of choice for high-accuracy measurements, in particular for purity determination,^5^ a broad range of applications require analysis of more dilute solutions. Direct analysis of these solutions is preferred to avoid cumbersome extraction and sample preparation procedures, reduce the associated errors, and increase throughput. This is often the case in natural products, metabolomics and metabonomic applications, for example. The use of nondeuterated solvents in ^1^H NMR requires solvent suppression as the presence of the large solvent peak leads to a limited dynamic range of the method and, in turn, limits its ability to detect compounds at lower concentrations. When solvent suppression is applied during NMR acquisitions, it may be among the largest sources of variability in the results and consequently its use is often limited in high-accuracy applications.^6^

The plethora of different methods for solvent suppression complicates the choice of the most suitable approach. It has become a common practice to adopt the first increment of a 1D nuclear Overhauser enhancement spectroscopy sequence with presaturation (1D-NOESYpr) as the method of choice across various areas.^7–9^ Despite being named after its 2D version, the use of this sequence for solvent suppression does not rely on any nuclear Overhauser effect (NOE) buildup. Instead, it employs presaturation to suppress the main solvent signal and the phase cycling of three subsequent pulses to achieve a *z*-filtering volume selection in what is called suppression of faraway water.^10,11^ While this sequence has become the gold standard for suppression in metabolomics, it has been suggested that other sequences offer superior performance.^12^ When using the 1D-NOESYpr sequence, one has to define the duration and field strength of the presaturation pulse and an appropriate mixing time. All presaturation sequences depend strongly on the careful setting of the carrier frequency which often has to be manually optimized and can lead to loss of performance or inconsistencies between samples when the solvent signal frequency varies slightly. Purge NMR has been proposed as an alternative *z*-filtering sequence potentially outperforming 1D-NOESYpr in some applications yielding an effective and straightforward method for selective suppression.^13^ The duration and field strength of the presaturation, however, may still have a huge impact on results and several different values for these parameters can be found in the literature for very similar applications, reflecting the complexity of the optimization process for presaturation sequences and their sample-dependent performance. Furthermore, exchangeable protons may not be observed when presaturation is used and quantitation of protons with slower exchange rates may be impaired by the use of such sequences. Several alternative pulses and pulse sequences are now available aimed at delivering better suppression characteristics such as the Perfect-Echo W5 (PEW5),^14^ Robust5 ^15^ and, more recently, pulses developed using modern Genetic Algorithms (Jump-and-return Sandwiches (JRS))^16^ and Artificial Intelligence (Water Irradiation Devoid (WADE)).^17–19^ In this paper, we conduct a full assessment of the strengths and weaknesses of these suppression sequences in comparison with the conventionally adopted methods and evaluate their ability to produce high-accuracy results in the presence of nondeuterated solvents. Measurements of the excitation profiles of different sequences were used to determine how close from the suppression range a signal can be without losing quantitative information. Optimized sequences were developed using WADE pulses with a Perfect-Echo, as well as optimized JRS pulses that can be used in higher-field instruments where other alternatives may fail either due to the very strong radiation damping caused by 100% H_2_O as a solvent or due to limited bandwidth of the pulses. Finally, we estimate measurement uncertainty for qNMR measurements in the absence of deuterium enriched solvent (no-D qNMR) using an internal standard and compare these estimates to measurements of the same sample in a traditional internal standard qNMR experiment.

## Experimental Section

### Chemicals and Materials

Deuterium oxide (99.9% atom D), sucrose (99.5%), d-glucose monohydrate (96%), and kainic acid monohydrate (99%) were purchased from Sigma–Aldrich. Vanillin was from Isofar. Lactose monohydrate was from Chem Service, and its purity was determined internally by a mass balance approach. Maleic acid certified reference material (CRM) 8792.0001 was from the Brazilian National Institute of Metrology, Quality and Technology (Inmetro).

### Sample Preparation for NMR Solvent Suppression Optimization

d-glucose (5.62 mg – 14.2 mM), lactose (5.39 mg – 7.5 mM), sucrose (5.79 mg – 8.5 mM), vanillin (5.00 mg – 16.4 mM) and internal standard (maleic acid – 2.20 mg – 9.5 mM) were prepared in triplicate into 10 mL screw cap vials; 1.7 g of water and 0.3 g of D_2_O were weighed in the same vial. This solution was vortexed for 1 min and transferred to a 5 mm high-throughput NMR tube. Weighing was performed on a calibrated Mettler XP205 balance with 0.01 mg resolution.

### Sample Preparation for no-D qNMR Solvent Suppression Optimization

Kainic acid (4.010 mg – 17.3 mM) and internal standard (maleic acid, 4.441 mg – 38.3 mM) were weighed into a 10 mL screw cap vial; 1 mL of type 1 water was added and the solution was vortexed for 1 min and transferred to an NMR tube. Reference samples were prepared using 4.723 mg of kainic acid and 5.108 mg of maleic acid with D_2_O as solvent. Weighing was performed on a calibrated Mettler XP6 balance with 0.001 mg resolution.

### Instrumentation

All NMR experiments were performed at 20 ± 0.1 °C on three different systems. The initial evaluation of the 1D-NOESYpr sequence and the intermediate precision test to assess the transference of the Robust5 sequence were done using a Bruker Avance III HD spectrometer with an Ascend 500 magnet operating at 11.74 T equipped with a Prodigy CPP TCI inverse cryoprobe with 65.7 G/cm maximum gradient strength. The two other systems were: a Bruker Avance III spectrometer with a Ultrashield 500 magnet operating at 11.74 T equipped with a PA TXI inverse probe with 55 G/cm maximum gradient strength, and an Avance III spectrometer with a Ultrashield Plus 700 magnet operating at 16.44 T equipped with a CP TCI cryoprobe with 53 G/cm maximum gradient strength.

### General NMR Acquisition Parameters

Size of the FID 128k points, spectral width 30 ppm, excitation pulse lengths were calibrated at 90°, excitation offset in resonance with the solvent signal, repetition times were >10 times the longitudinal relaxation time constants (T1) for quantitative experiments and 15 s for the excitation profiles. T1 in quantitative experiments were determined using inversion–recovery sequences with relaxation delays of at least 30 s. For the determination of the excitation profiles, an NMR tube containing D_2_O was used and the excitation offset was swept in 0.05 ppm steps. The HOD signal was used to calculate the excitation profile. We assessed the following Bruker standard sequences: presaturation (zgpr), composite pulse presaturation (zgcppr), 1D-NOESYpr (noesypr1d), 1D-NOESYgppr (noesygppr1d), WET (wet), and PURGE (zgpurge). For presat and cppresat, a 20 s presaturation was used at an effective radio-frequency (RF) field strength of 50 Hz. 1D-NOESYpr and 1D-NOESYgppr used mixing times of 2 and 150 ms at 50 Hz for 4.5 s, according to Giraudeau et al.^7^ and 20 s. A mixing time of 2 ms for 35 s at 29.5 Hz, based on the work of Canlet et al.,^9^ was also applied. PURGE used a 4.5 s presaturation at 50 Hz. WET used 20-ms Gaussian shaped selective pulses. Initial data processing was performed using Bruker TopSpin 4.4.1 and all data processing for the quantitative analyses was undertaken in MestReNova 15.

### Measurement Uncertainty Estimation

Calculations were conducted in R (v. 4.4.1). Nonlinear least-squares regression was performed to determine T1 values and their associated standard deviations. Package ggpubr^20^ was used for normality tests and robustbase^21^ for robust statistics. Monte Carlo Simulations were performed using an in-house developed script in R base with 10 000 simulations.

## Results and Discussion

1D-NOESYpr is one of the most widely used sequences for solvent suppression in qNMR determinations.^7–10^ To assess the performance of this method, we used a gravimetrically prepared mixture of vanillin, lactose, glucose, and sucrose. The mixture was designed choosing compounds belonging to structural classes of interest in natural products and metabolomics that were readily available commercially at high purities, with signals of interest distributed over a broad range of chemical shifts. The Bruker standard pulse program noesygppr1d was used with one slight modification where the relaxation delay was split in two periods (D11 and D1). The first (D11) did not have an associated presaturation to allow full control of the repetition and presaturation times independently. This splitting was done to all presaturation-based pulse sequences used in this work, providing repetition times equal to or greater than 10× the T1 constants, regardless of the presaturation time used. Analysis of this test mixture used 15 s of presaturation within a total repetition time of 57.7 s (AQ = 3.27 s, D11 = 39.43 s and D1 = 15 s). The presaturation pulse RF field strength (γB_1_) was varied from 8 to 50 Hz and the analyte signals were integrated and normalized to 1 as the maximum signal area for each respective signal (Figure 1). The RF field strength for presaturation is a critical parameter that must be optimized manually on a sample-by-sample basis for all presaturation-based sequences. While values ranging from 29 Hz to 80 Hz have been reported as ideal conditions,^7,9,22,23^ significant signal losses occurred at any RF field strength above 12 Hz with our experimental sample, as shown in Figure 1. Quantitation of metabolites in synthetic urine has been reported with good accuracy using a 29.5 Hz RF field strength by Canlet et al.,^9^ where the optimized RF field strength resulted in a H_2_O peak matching the intensity of the most intense metabolite peak. On the other hand, Ravanbakhsh et al.^23^ have reported metabolite quantitation on biofluids including urine with a very similar preparation procedure and an RF field strength of 80 Hz.

**Figure 1:**
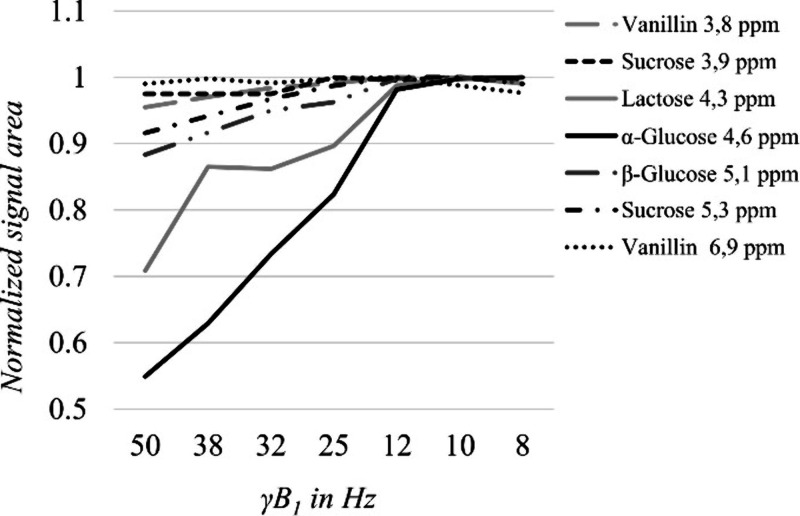
Effective RF field strength (γB_1_) versus normalized signal areas for different analytes using a NOESYpr sequence with 15 s of presaturation.

Therefore, it is clear that, to achieve appropriate accuracy, these sequences require sample-by-sample optimization, which makes their use challenging for routine applications with different types of samples. Another key issue is the limited dynamic range that may result from the use of these sequences. In this sample, the H_2_O peak was the most intense signal when RF powers of 8, 10, and 12 Hz were applied and would be the limiting factor in the setting of the receiver gain and, as a result, for the dynamic range of the experiment. For RF field strengths above 25 Hz where the solvent signal is no longer the limiting factor, there are already severe signal losses meaning there is no optimal condition for this sample considering both signal loss and dynamic range, even at a relatively high concentration.

This can be even more critical when protonated solvent is used. The limitation in dynamic range yielded by solvent signals in NMR is a known issue for several decades and restricts the application of qNMR for dilute samples. More efficient solvent suppression techniques are needed to make such samples accessible by qNMR.^24^ The limitations produced by the solvent signal in receiver gain were also clear in the no-D experiments with kainic acid and maleic acid. No-D qNMR experiments are important for the production of CRMs of compounds that are rare or difficult to obtain, enabling qNMR measurements on the same material that will be used in the production of the reference materials.^25^ Kainic acid was chosen due to its structural similarity with rare or difficult to obtain natural products that we use in producing CRMs. In these cases, no-D applications fully exploit the nondestructive nature of the technique as they can be used when one wishes to avoid H/D exchanges and excessive sample manipulation during preparation. Here we used the noesypr1d or the noesygppr1d with a 35 s presaturation period with 29.5 Hz. The resulting spectra still show a water signal close to 2 orders of magnitude more intense than the second most intense signal (maleic acid). The use of a higher presat RF field of 100 Hz during 15 s with 1D-NOESYpr and PURGE did not lead to an efficient suppression to the point of a water signal comparable to the analyte signals (Figure 2). Essentially, this means that the dynamic range of the analysis would potentially be affected by the presence of this large residual solvent signal, in the analysis of more dilute samples. The Robust5 sequence is presented for comparison where the water residual signal is 55 times less intense than the maleic acid signal. Such an efficient solvent suppression enables analyses of much more dilute samples and helps bridging the detectability gap between NMR and other analytical techniques.^24^

**Figure 2:**
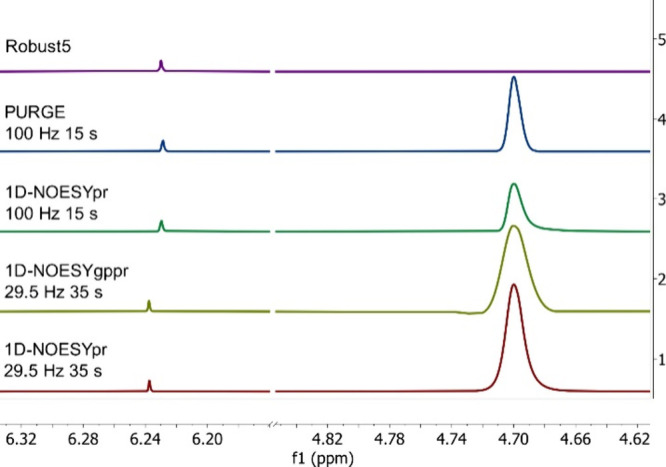
no-D NMR spectra at 500 MHz with different suppression schemes for a sample containing maleic acid and kainic acid (approximately 4 mg/mL each), using pure H_2_O as the solvent, demonstrating the efficiency in reducing the residual water with each scheme. The intensities of the spectra were normalized using maleic acid and the region between 4.85 and 6.15 ppm was omitted to improve visualization.

Commercial kainic acid monohydrate was prepared in D_2_O with a maleic acid CRM as internal standard to determine its purity using a reference qNMR method previously validated by some of us.^5^ The measured T1 for maleic acid was 5.4 s while for kainic acid the values were 1.16 s (1.64 ppm), 0.66 s (2.36 ppm), 1.3 s (2.98 ppm), 0.54 s (3.33 ppm), 0.64 s (3.54 ppm), 2.49 s (4.15 ppm), 0.71 s (4.65 ppm) and 0.92 s (4.93 ppm). The longest T1 (maleic acid, 5.4 s) was used to define the repetition times with all quantitative analyses performed using a repetition time of at least 54 s. The purity of the sample was determined as 1000 mg/g (100% w/w) with an expanded u ncertainty (*k* = 2) of 0.62%. Accurately prepared gravimetric samples of this kainic acid using the XP6 balance–resolution of 0.001 mg–were then used to assess accuracy of the quantitations with solvent suppression.

Using the internal standard, we calculated the mass of analyte present in the tube using eq 1:

$$m_{a}=\frac{I_{a}N_{IS}{\mathrm{MM}}_{a}m_{IS}P_{IS}}{I_{IS}N_{a}{\mathrm{MM}}_{IS}}$$ 1

where *m* is the mass (in mg), *I* is the area of the NMR signal, *N* is the number of equivalent hydrogen atoms for the NMR signal, MM is the molar mass (in g/mol), *P* is the purity (in g/g), the subscript *a* refers to the analyte, and the subscript *IS* refers to the internal standard. To assess trueness, we used the relative bias percentage, calculated as *b*_%_ = (*m*_*a*_ – *m*_*a.theor*_)/*m*_*a.theor*_ × 100, where *m*_*a.theor*_ was the mass of analyte added gravimetrically to the sample. A set of three signals from kainic acid were chosen to calculate the biases due to their T1 and chemical shift distributions.

The data presented in Chart 1 reveal that 1D-NOESYpr and PURGE outperform the other standard sequences by having smaller biases, as expected. This also explains why the two sequences are the most widely applied in the literature. Although it has been reported that 1D-NOESYpr with a mixing time of 150 ms may lead to accurate results,^7^ it can be seen that, in our case, it only provides accurate results for the kainic acid signal at 4.15 ppm and extremely poor results for the other two signals assessed. This is due to the fact that these two signals have short relaxation time constants and therefore their loss due to relaxation during a 150 ms mixing period is considerable. This limitation highlights yet another parameter that must be optimized on a sample-by-sample basis if 1D-NOESYpr is used. In all other experiments with 1D-NOESYpr, we used an alternative mixing time of 2 ms to avoid those losses. WET shows excellent accuracy for signals farther from the suppression range but suffers from very poor accuracy closer to the suppression range, even with a 26.25 ms selective pulse, which combined with an intense water residual signal greatly limits its applicability. On a no-D qNMR setting, the limitations in dynamic range for PURGE and 1D-NOESYpr are significant and preclude the use of the technique for quantitation of natural products in dilute solutions. Therefore, as an alternative, we explored the use of Robust5,^15^ which is based on an excitation sculpting version of the “perfect echo” WATERGATE (WATER-suppression by GrAdient-Tailored Excitation)^14^ with the addition of alternating gradients and of a time correction on the W5 pulse elements that improves the bandwidth of the excitation.^26,27^ Unlike most of the other solvent suppression approaches, the excitation sculpting sequences using double-echo such as Robust5 (as well as PEW5 and JRS8 that will be discussed) readily produce outstanding results with no need for optimization, which is an extremely important feature for their implementation in routine and automated spectroscopy applications. A comparison between a kainic acid no-D sample spectrum obtained with this sequence and one obtained with the 1D-NOESYpr is presented in Figure 3.

**Chart 1:**
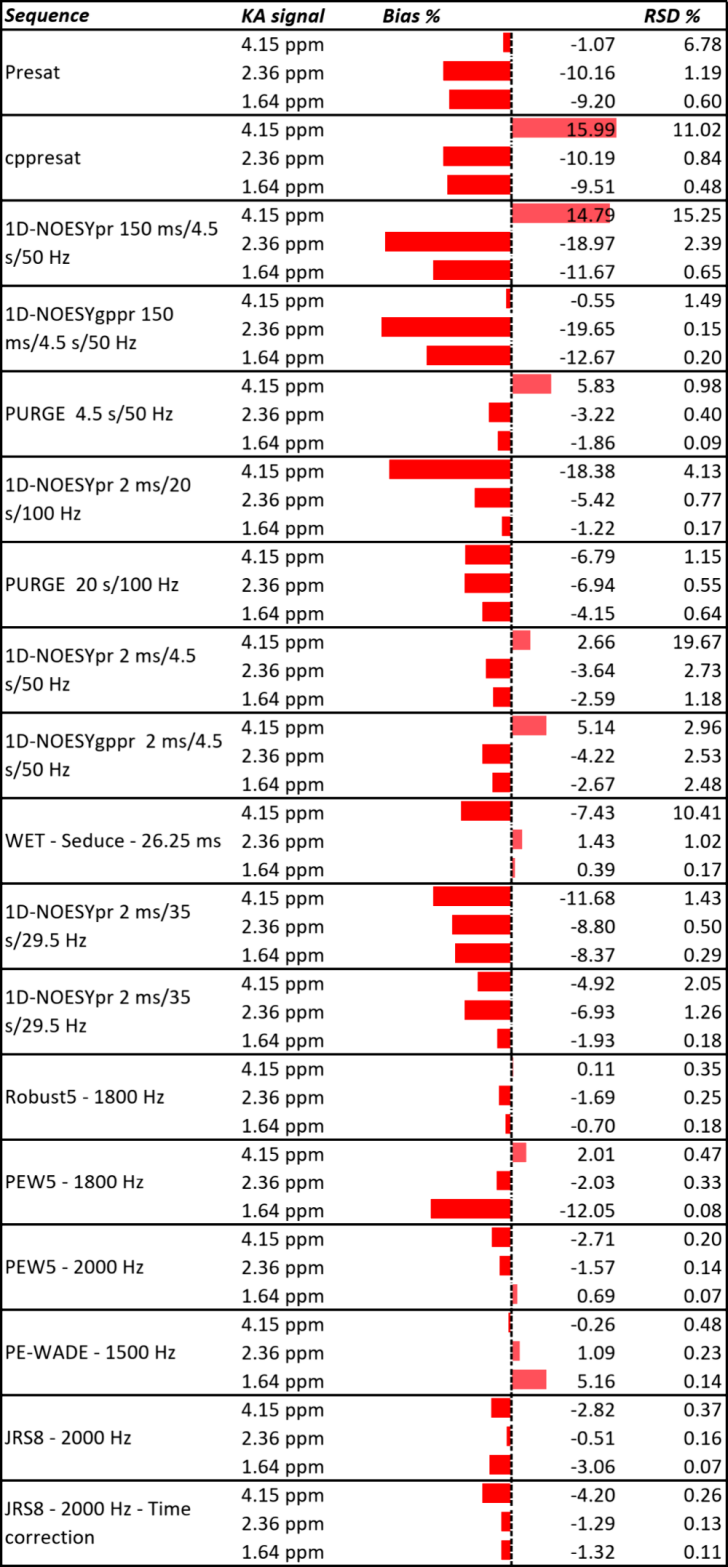
Relative Bias (*b*_%_) Observed for Three Kainic Acid Signals and Their Repeatabilities (RSD, *n* = 5) with Different Suppression Sequences at 500 MHz^a^

a All NOESY sequences refer to 1D-NOESYpr.

**Figure 3:**
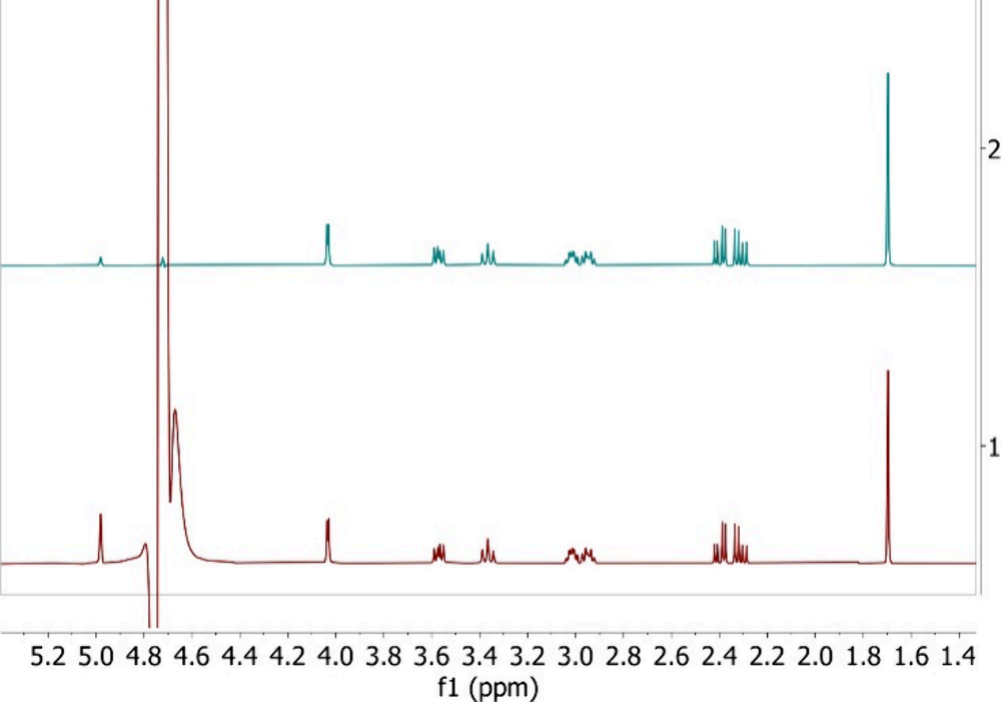
Comparison of no-D spectra acquired at 500 MHz for kainic acid with different suppression schemes. The green spectrum was acquired using Robust5 with cnst10 = 1800 Hz and the red spectrum using 1D-NOESYpr with 2 ms mixing time and 35 s of suppression at 29.5 Hz effective RF field.

As Robust5 is based on the W5 binomial-like element, the only parameter that needs to be set in addition to a normal 1D acquisition is the interpulse delay of the W5 element that controls the width of the suppression range and the position of the secondary suppression notches that result from the application of such scheme. Because Robust5 uses the time-corrected W5 element,^27^ the interpulse delays are not all equal and their calculation is implemented by the use of a constant in the sequence (cnst10) corresponding to the distance to the next null point and is used to calculate the appropriate corrected delays. In this case, we applied a cnst10 of 1800 Hz and performed an experiment to confirm that the excitation profile matched our expectations. This experiment was conducted by acquiring a series of 200 spectra using D_2_O with varying carrier frequencies (Figure 4) and confirms the expected excitation profile with two additional suppression notches separated from the central suppression by 1800 Hz. The suppression band is 600 Hz wide, which means that analyte peaks beyond ±300 Hz from the solvent signal can be quantitated with high accuracy, since the excitation efficiency is over 97% in that range. In a 11.74 T (500 MHz for ^1^H) instrument, such as the one used for these experiments, this means that signals ±0.6 ppm from the solvent can be used for high-accuracy quantitation. In fact, the data presented (Chart 1) show that use of Robust5 with the 1800 Hz cnst10 leads to a bias of only 0.11% for the analyte signal at 4.15 ppm.

**Figure 4:**
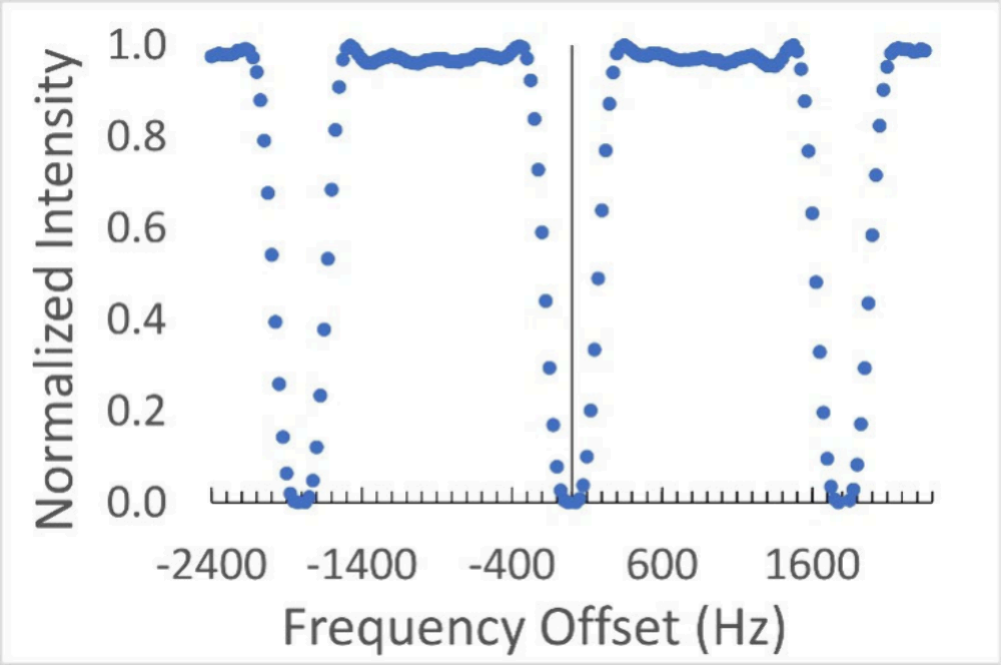
Robust5 excitation profile using cnst10 = 1800 Hz on a D_2_O sample. The offset presented at the *x*-axis is equal to ν_(D_2_O)_-ν_1_.

The binomial-like sequences (Robust5, PEW5, and JRS8) led to measured signal intensities 28–50 times higher with >5 times better signal-to-noise ratio, when compared to 1D-NOESYpr, while with the WADE-based sequence showed ∼15 times more-intense signals. This increased detectability reduces the gap in limits of detection between NMR and other techniques and may pave the way for trace analyses using qNMR. The main drawback with these sequences is the presence of the secondary notches which limits the use of signals farther from the suppression. A null at ±1800 Hz is also observed with the same width as the central suppression band, meaning that the usable regions of the spectrum for high-accuracy quantitation in this case span from approximately 1.7 ppm to 4.1 ppm and from 5.3 to 7.7 ppm. By increasing the cnst10 (or decreasing the interpulse delays), the secondary notches can be pushed further away with an associated increase in the width of the notches. Thus, it is necessary to choose interpulse delays that lead to appropriate excitation of the peaks for quantitation. If no signals of interest are present close to the solvent peak, then a shorter delay (larger cnst10) can be used to push the notches out of the spectrum. Excitation profiles for several different delays are presented in the Supporting Information as an illustration of this effect.

To fully understand the potential of similar sequences we used a general excitation sculpting scheme based on the Robust5 sequence depicted in Figure 5. The selected sequences yield solvent suppression using the excitation sculpting version of a double gradient echo sequence with a selective 180° element leading to a 180° rotation in most spectral resonances, but with zero net flip angle at the solvent resonance. Transverse magnetization is dephased by the first gradient pulse and, in order to be rephased by the second gradient pulse, it must undergo an effective 180° rotation in the middle of the gradient echo.

**Figure 5:**
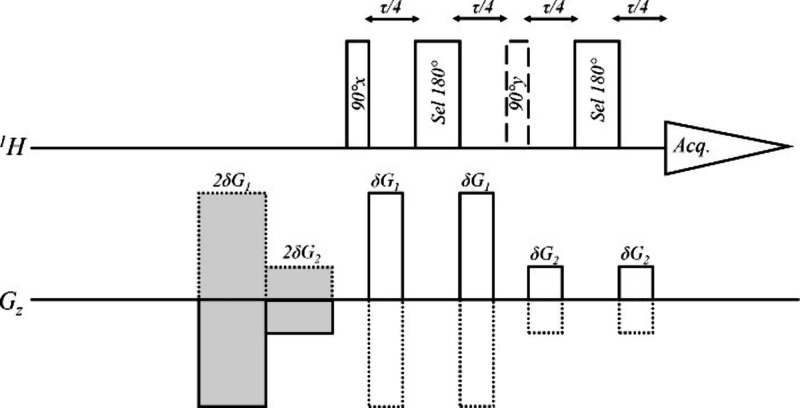
General structure of the solvent suppression pulse sequences used. The dotted lines represent alternating gradients; The construction of the sequences using this basic structure is (a) Robust5: time-corrected W5 elements used as the selective 180° pulses. G1 and G2 amplitudes of 53% and 9.14% (equal to 30.25 G/cm and 5.03 G/cm in the TXI probe, 29.15 G/cm and 4.84 G/cm in the CP TCI probe and 36.14 G/cm and 6.00 G/cm in the CPP TCI probe); (b) PE-WADE: WADE shaped pulses used as the selective 180° pulses. G1 and G2 amplitudes of 34% and 22% (equal to 18.7 G/cm and 12.1 G/cm in the TXI probe, 18.02 G/cm and 11.66 G/cm in the CPP TCI probe and 22.34 G/cm and 14.45 G/cm in the CPP TCI probe); (c) JRS8: gradients shaded in gray and the 90°_*y*_ pulse were omitted and a time-corrected version of the JRS8 pulse used as the selective 180° pulses. G1 and G2 amplitudes of 34% and 22%; (d) PEW5: the gradients shaded in gray were omitted and a regular version of the W5 element used. G1 and G2 amplitudes of 34% and 22%. The length δ of the gradients was 500 μs with a 200 μs stabilization delay.

Three decades ago, it was demonstrated that use of a second independent gradient echo improves the phase properties of this sequence producing what is called excitation sculpting.^28^ The addition of the quadrature 90° pulse in the middle of the sequence yields the perfect echo which offers refocusing of J coupling evolution resulting in the ability to use longer selective pulses without the intrusive effects of J modulation.^14^

The W5 element for inversion was first described in 1998,^26^ and several years later, Wang and co-workers^27^ noticed a distortion in those excitation profiles due to the chemical shift evolution during the RF pulses that produced significant effects in higher field instruments and proposed the alternative time-corrected version of W5 that is implemented in Robust5. The comparison of Robust5 and PEW5 data with a 1800 Hz separation between nulls (Chart 1) reveals the effect of the time correction in providing a flatter excitation profile closer to the secondary notches. PEW5 clearly loses some of the signal at 1.64 ppm as a result of the decay in excitation that was reported in that work.

In our experiments using a 16.44 T (700 MHz) system equipped with a cryoprobe, we observed phase anomalies in the signal at 1.64 ppm. We suspected this profile resulted either from radiation damping or due to imperfections in the W5 profile described above not being completely corrected. Increasing G1 amplitude to the maximum of the probe did not improve the profile, which would be expected if this distortion was due to radiation damping. On the other hand, changing the excitation profile by using different cnst10 values (between 1800 and 5100 Hz) had more impact on the phase profile of the signals, but did not lead to a full correction indicating that the pulse element itself was causing the issue. To confirm, we used a W5 sequence without the perfect echo pulse (Bruker sequence zggpw5) and the phasing issues were completely solved. However, severe J modulation was observed proving that the phasing issues are a remnant of the W5 element that get corrected by excitation sculpting and that the perfect echo impairs this correction. The use of the zggpw5 sequence for quantitation, however, is not possible as the huge J modulation artifacts significantly impact the measured signal areas. JRS and WADE pulses were developed using modern optimization algorithms, both aiming at improving the excitation profiles while maintaining or improving the noninversion region for the solvent resonance. We implemented the WADE and the JRS pulses, as shown in Figure 5, to evaluate them as alternatives to Robust5 when such phasing issues are observed and applied these sequences to similar no-D qNMR samples to assess their performance. These sequences provide excellent suppression performance with residual solvent signals comparable to those of Robust5 (see images in the Supporting Information for a direct comparison) and good quantitative performances shown in Chart 1.

The JRS pulses are incompatible with the perfect echo as they generate chemical-shift dependent phases. Such phase effects are corrected by the use of the excitation sculpting but preclude the use of the quadrature 90° pulse to refocus J modulation. The JRS family of pulses was designed to provide superior performance with more selective noninversion bands. Hence, a JRS sequence with 8 pulses provides selectivity comparable to the W5 with its 10 pulses^16^ with a reduced duration of roughly 3.8 ms for the JRS8 element with 0.554 ms delays compared to 4.9 ms of the W5. This reduced duration was enough to avoid the J modulation artifacts even without the use of the Perfect Echo. Pellizzari and co-workers observed a similar effect with the use of JRS8 in benchtop instruments, where much longer interpulse delays are needed.^29^ In fact, our experimentally determined excitation profiles show that the JRS8 sequence results in a suppression band that is even narrower than that of the W5 based sequences (Robust5 and PEW5). It is important to note that all our JRS8 quantitative experiments were performed using a time-corrected version of the JRS8 where the interpulse delays were adjusted as reported by Wang et al.^27^ for the W5 sequence. Although the effect of this correction seems to be smaller in JRS8, compared to its effect in W5 sequences, our excitation profile experiments show that without the correction, the secondary notches appear closer to 1750 Hz instead of the expected 1800 Hz.

The sequence using WADE pulses is compatible with the perfect echo in the excitation sculpting setting presented in Figure 5 and the WADE pulse elements optimized for this work had durations that were comparable to those of the binomial-like and JRS elements already discussed. The WADE pulse element with RF amplitude equal to 1500 Hz used with the 500 MHz system for the data reported in Chart 1 had a length of 5.7 ms while the one used in the 700 MHz system with an RF amplitude of 2083 Hz lasted 4.1 ms. On the 500 MHz system, with the longer pulse in particular, the ability to use the perfect echo is an important feature as it leads to spectra that are free from J modulation artifacts. The quantitative performance of the sequence is inferior when compared to Robust5 but shares the very good suppression performance of Robust5 and JRS8 leading to a small residual solvent peak that offers improved dynamic range for qNMR acquisitions especially in a no-D setting. While WADE does not show secondary suppression notches, its excitation profile shows increased values toward the edge of the spectral range, meaning there is some loss close to the region of the inversion that explains the positive bias in results of the signal at 1.64 ppm in Chart 1. Robust5 produces the most accurate results in general but when it fails, as is the case with the 700 MHz system used in this work, JRS8 and PE-WADE lead to results that are almost as accurate and offer good alternatives to the analyst.

We also investigated the ease of implementation and reproducibility of the Robust5 sequence by analyzing the same kainic acid sample in another laboratory on an 11.74 T (500 MHz) instrument equipped with a prodigy cryoprobe. Similar results were obtained without any need for adjustments in the sequence, highlighting the ease of implementation and use of the sequence (data not shown).

The ability to determine T1 constants relatively accurately is important to produce accurate qNMR results. Although sufficient material was available to measure T1 in samples prepared in D_2_O and use that as a surrogate to define the repetition times for the samples measured in no-D qNMR (pure H_2_O), we also wanted to implement an approach that would allow the determination of T1 directly in no-D samples. To achieve that, we designed an inversion–recovery pulse sequence with the addition of the perfect echo W5 block after the 90° pulse. Since the duration of this suppression block is significant, a 1D version of the inversion–recovery sequence could lead to biased T1 results due to the relaxation that occurs during suppression. For this reason, we implemented this sequence only as a pseudo-2D sequence (Figure 6).

**Figure 6:**
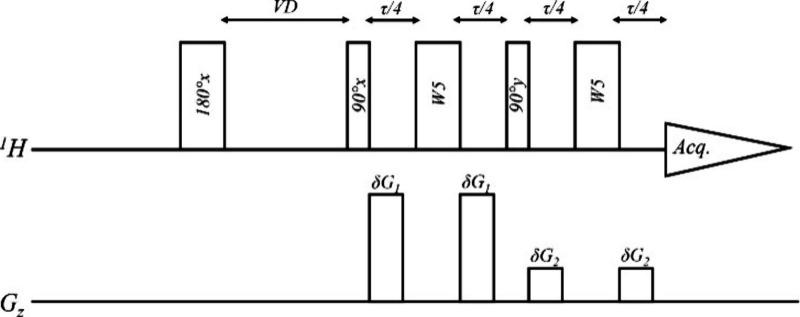
Inversion–recovery sequence with the perfect echo W5 block for solvent suppression. A pseudo-2D implementation was done using a variable delay (VD) and the regular W5 pulse elements.

We were able to obtain spectra with minimal residual solvent signals and an excellent adjustment to the exponential model for relaxation. The T1 constants determined for the maleic acid + kainic acid sample in H_2_O were as follows: for maleic acid, 3.85 s; for kainic acid, 1.16 s (1.64 ppm), 0.68 s (2.36 ppm), 1.14 s (2.98 ppm), 0.53 s (3.33 ppm), 0.56 s (3.54 ppm) and 1.03 s (4.15 ppm). It was not possible to measure T1 values for the signals at 4.65 and 4.93 ppm, because they are within the suppression band.

The T1 values measured in water are in close agreement to the ones measured in D_2_O, with the exception of the maleic acid signal and the 4.15 ppm kainic acid signal, both of which show shorter T1 values. The most significant difference was observed for the 4.15 ppm signal and may be partially due to radiation damping as this signal is closer to the water signal. In any case, these T1 values are the ones that most effectively describe the relaxation process of the sample in a no-D setting and being able to accurately measure them using this sequence enables the use of appropriate repetition times for no-D qNMR. Analogously, we successfully implemented the binomial-like suppression methods in Carr–Purcell–Meiboom–Gill (CPMG) pulse sequences for T2 measurements, showcasing the ease of implementing these methods in different sequences. Since these sequences are longer than the normal pulse-acquire sequence, relaxation losses could lead to biases. Although the results in Chart 1 show that this is not an issue, because we are able to obtain values that are closely matched to the gravimetric reference values, one could use correction approaches similar to the recently published EXQUISITE method to account for those losses, if even better trueness is needed.^30^

Another additional source of variability is the result of the fluctuations in the excitation profile. In single-pulse 1D ^1^H NMR spectra, the excitation profile is sufficiently flat over the chemical shift range and off-resonance effects are almost negligible. Therefore, magnetization will only be influenced by the stochastic nature of data acquisition, which ultimately gets reflected in the repeatability component of the measurement. The excitation profiles for the multipulse sequences used for suppression are not perfectly flat, which can be a source of error in measurements that has to be accounted for in uncertainty estimation. We defined the usable spectral region based on the width of the suppression notch for each suppression sequence. These regions were selected visually as close as possible to the inflection before the suppression notches, which resulted in the selection of data points where excitation was above 97% for all sequences, except for the JRS8- and WADE-based sequences. All the data points within the usable regions were plotted and a Shapiro–Wilk test and a Q–Q plot applied to check for normality. Since the assumption of a normal distribution was not valid for any of the distributions, we used a robust descriptor of the location and the uncertainty of the magnetization in these areas. We used the Huber-M estimator of location with median absolute deviation (MAD) scale^31^ and the Q_*n*_ estimator^32^ as the corresponding uncertainty. The usable areas are ultimately the areas in which these uncertainty estimates apply, meaning that if signals in these regions are used, an uncertainty allowance of that magnitude is expected to be included in the results. The estimates are presented in Table 1, and it can be seen that higher fluctuations in excitation with JRS8 and WADE were reflected in higher measurement uncertainty estimates. The table also summarizes the width of the suppression notches observed in each sequence, highlighting that the binomial-like sequences offer more selective suppression with narrower notches.

**Table 1:** Measurement Uncertainty Estimates with Respect to Signal Intensity Profiles in Different Suppression Sequences

sequence	suppression width (Hz)	usable range end points^a^ (Hz)	relative standard uncertainty associated with excitation^b^ (%)
zgpr	800	400	0.39
noesypr1d	900	450	0.78
Robust5 1800 Hz	600	300–1500	1.03
Robust5 2400 Hz	800	400–2000	1.09
PEW5 1800 Hz	600	300–1450	1.07
PEW5 2400 Hz	800	400–2000	1.21
JRS8 1800 Hz	500	250–1550	2.71
JRS8 2400 Hz	650	325–2075	2.73
PE-WADE	650	325	3.45

a The usable range applies to both sides of the main suppression notch. When only one value is reported, a secondary notch is absent.

b The uncertainties are presented as relative to the robust estimation of the excitation in the usable region.

As expected, the less complex the pulse sequence is, the smaller the uncertainty associated with the signal intensity profile. The W5-based sequences show slightly higher uncertainties, compared to noesypr1d, but this uncertainty is outweighed by better repeatability uncertainty, due to the much greater signal intensities enabled by these much-more-efficient suppression schemes. Finally, JRS8- and WADE-based sequences show much higher uncertainties associated with the signal intensity profiles, but remain adequate alternatives for trace analyses in no-D conditions for higher field instruments or for cases where the W5 inversions may fail. All other measurement uncertainty components taken into account for measurements with and without suppression are presented in the Supporting Information, as well as the R scripts for Monte Carlo Simulation. While the uncertainty budgets for internal standard qNMR are well described and understood,^5^ additional uncertainty allowances arising from the use of solvent suppression have never been described, to the best of our knowledge. The uncertainties associated with the signal intensity profiles were accounted for in Monte Carlo Simulations. This led to a final standard uncertainty estimate between 1.52% and 1.77% for the Robust5 results. For comparison, results with the same sample using D_2_O and no solvent suppression led to uncertainties ranging from 0.71% to 0.89%. Thus, the use of Robust5 solvent suppression nearly doubled the achievable measurement uncertainties. This increase in measurement uncertainty from solvent suppression has been frequently neglected in the literature on qNMR measurement. While some potential impacts of the use of presaturation-based sequences such as saturation transfer and partial suppression of the signals are not quantifiable, the pulse sequences used in this work allow a realistic estimation of the associated uncertainty components.

## Conclusions

A range of pulse sequences were assessed for solvent suppression in qNMR and demonstrated their limitations in achieving high accuracy results, especially in the absence of deuterated solvents. The suppression produced by presaturation-based sequences is inappropriate for very low concentrations of analytes. Although more research on dilute samples is needed, binomial-like sequences may help bridge the gap in sensitivity between NMR and other techniques by enabling better signal-to-noise ratios and lowering limits of detection.

Based on signal intensity profiles, the binomial-like sequences offer a usable spectral range that extends to as close as 300 Hz from the suppressed solvent in no-D experiments with an associated uncertainty as low as 1.03%. The usable range in these sequences can be tailored to specific needs by manipulating the interpulse delays offering great flexibility for achieving high-accuracy results in most situations. The implementation of two pulse sequences based on modern pulses—namely, JRS8 and WADE—and a comparison of their performance with the sequences based on the W5 pulse show that, although these did not prove to be as accurate as the W5-based sequences, they are appropriate alternatives in cases where W5 pulses fail, especially at ultrahigh fields.

The development of an inversion–recovery sequence for T1 measurement using the perfect echo W5 element was presented. Accurate T1 measurements are of utmost importance to qNMR, and the ability to determine these values in the actual samples to be measured even without deuterated solvents increases the accuracy and reliability of the technique. Finally, we demonstrated that robust evaluation of uncertainty allowances related to the excitation profile are needed to provide a realistic uncertainty budget for qNMR using solvent suppression. The use of Robust5 roughly doubles the measurement uncertainty achieved with deuterated solvent but still leads to high accuracy results with relative standard uncertainties ranging from 1.54% to 1.77% using an internal standard method. With regard to the binomial-like sequences, the limitations imposed by the existence of the secondary notches can be overcome by adjusting the interpulse delay so that the notches are not in the usable range of the spectrum. These notches can also be useful for suppressing secondary peaks from additives in the solution such as formic acid while retaining excellent quantitative performance.

## Supplementary Material



## Notes

Excitation profiles for different sequences used in the work (Figures S1–S10); comparison of the suppression performance of Robust5, JRS8, and PE-WADE (Figure S11); Case-effect diagram used for measurement uncertainty estimation (Figure S12); Uncertainty budgets with and without suppression (Tables S1 and S2); Relative contributions to measurement uncertainty (Figures S13 and S14); R script used for uncertainty estimation with Monte Carlo Simulation;Pulse programs (Bruker format) (PDF)
