# Supplementary material for: High-Accuracy Quantitative Nuclear Magnetic Resonance Using Improved Solvent Suppression Schemes
Source: Anal Chem. 2025 Sep 25;97(39):21240–8. doi: 10.1021/acs.analchem.5c01139 (PMC12509184; doi:10.1021/acs.analchem.5c01139)
Supplement: Supplementary file 1 [file ac5c01139_si_001.pdf]

Supporting Information

High Accuracy Quantitative Nuclear Magnetic Resonance using Improved Solvent Suppression Schemes

Bruno C Garrido[a]*, Lucas J Carvalho[b], Ian W. Burton[c], Pearse McCarron[a]

[a] Metrology Research Centre, National Research Council of Canada, 1411 Oxford St, Halifax, NS, Canada, B3H 3Z1

[b] Organic Analysis Laboratory, National Institute of Metrology, Quality and Technology, Avenida Nossa Senhora das Graças, 50, Duque de Caxias, RJ Brazil

[c] Aquatic and Crop Resources Development, National Research Council, 1411 Oxford Street, Halifax, B3H 3Z1, NS, Canada

* Email: Bruno.garrido@nrc-cnrc.gc.ca

Table of Contents

Page S2: Figures S1-S10 - Excitation profiles with different inter-pulse delays (based on cnst10 – see main text)

Page S6: Figure S11- Comparison of the suppression performance of Robust5, JRS8 and PE-WADE.

Page S7: Figures S12-S14 and Tables S1 and S2: Measurement Uncertainty estimation.

Page S9: R script for measurement uncertainty estimation using Monte Carlo Simulation.

Page S10: JRS8 pulse program.

Page S12: PE-WADE pulse program.

Page S14: T1 measurement pulse program.

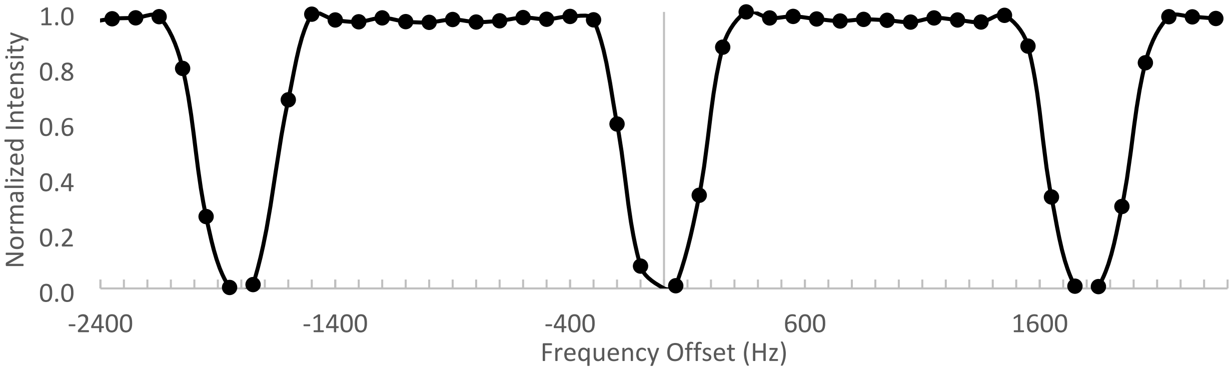

Figure S1 - Excitation profile of Robust5 at 500 MHz using cnst10 = 1800 Hz on a D_2O sample. The offset presented at the x-axis is equal to $\nu_{(D2O)}$-ν_1.

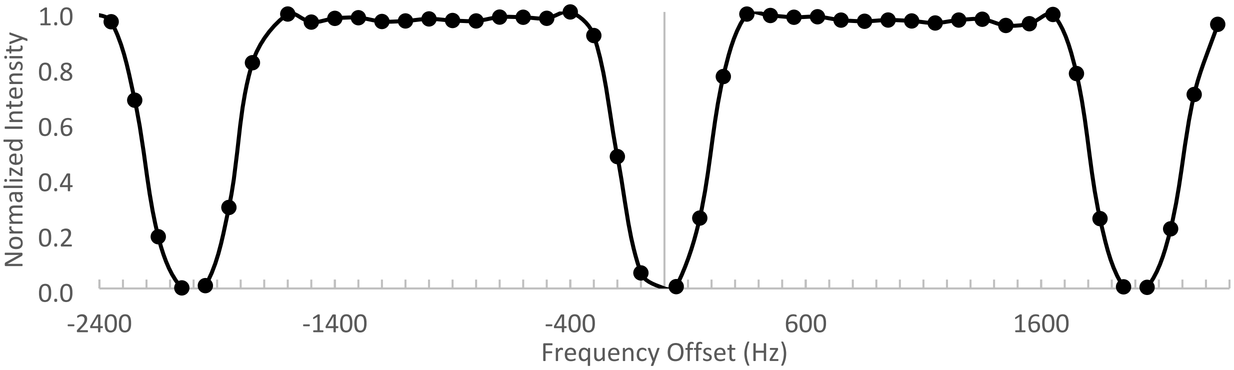

Figure S2 - Excitation profile of Robust5 at 500 MHz using cnst10 = 2000 Hz on a D_2O sample. The offset presented at the x-axis is equal to $\nu_{(D2O)}$-ν_1.

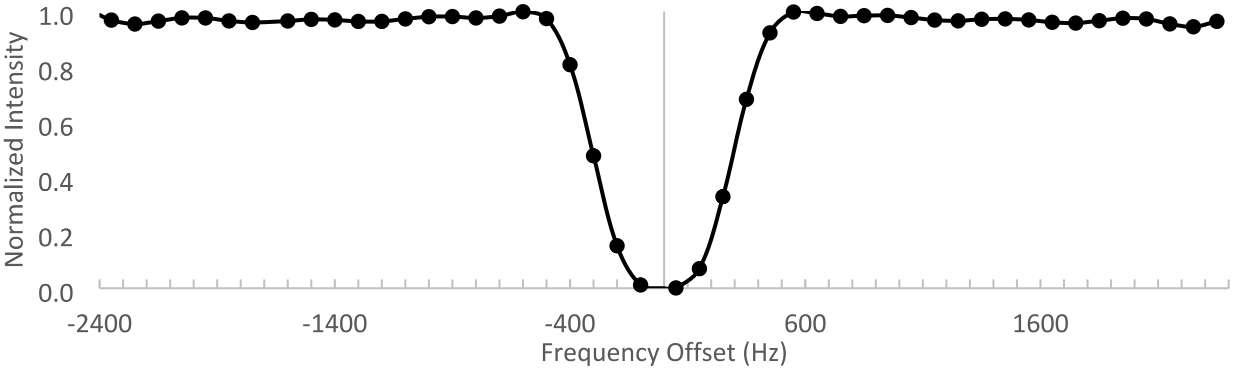

Figure S3 - Excitation profile of Robust5 at 500 MHz using cnst10 = 3000 Hz on a D_2O sample. The offset presented at the x-axis is equal to $\nu_{(D2O)}$-ν_1.

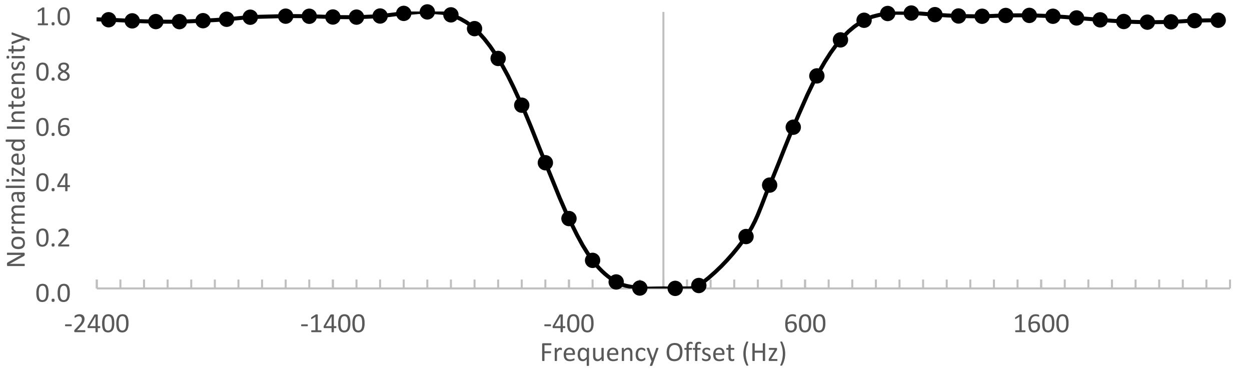

Figure S4 - Excitation profile of Robust5 at 500 MHz using cnst10 = 5100 Hz on a D_2O sample. The offset presented at the x-axis is equal to $\nu_{(D2O)}$-ν_1.

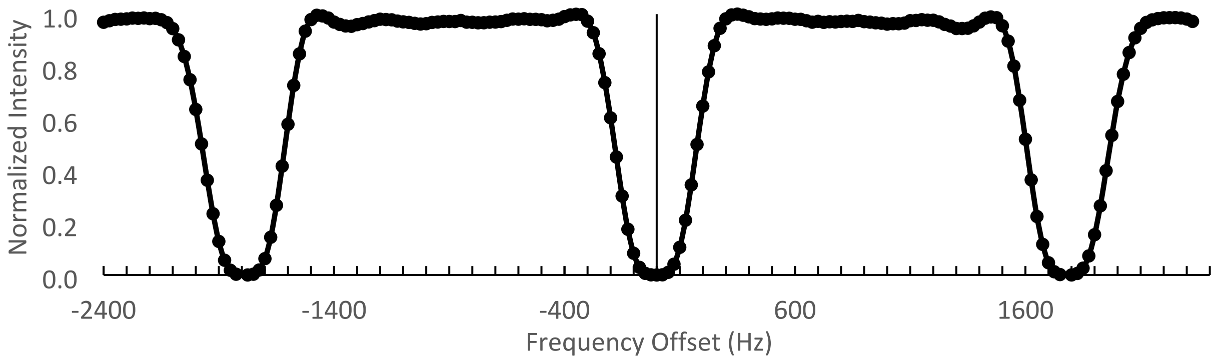

Figure S5 - Excitation profile of PEW5 at 500 MHz using an inter-pulse of 0.556 ms (equivalent to 1800 Hz cnst10) on a D_2O sample. The offset presented at the x-axis is equal to $\nu_{(D2O)}$-ν_1.

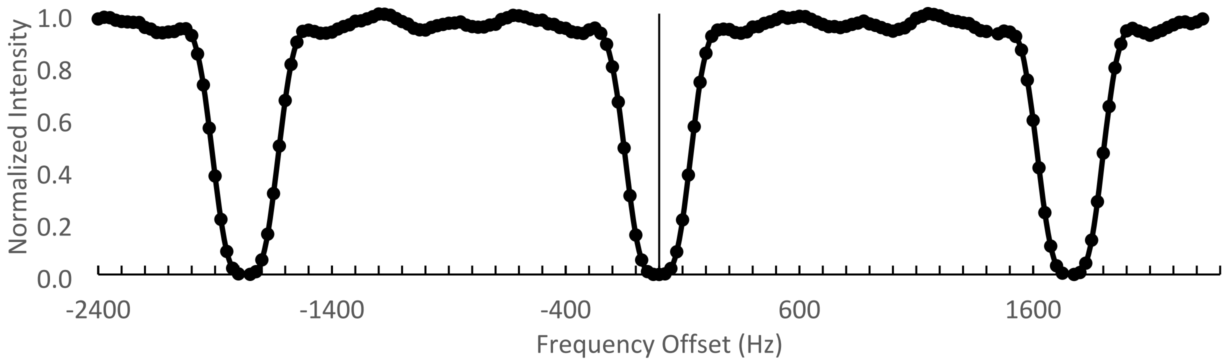

Figure S6 - Excitation profile of JRS8 at 500 MHz using an inter-pulse of 0.556 ms (equivalent to 1800 Hz cnst10) on a D_2O sample. The offset presented at the x-axis is equal to $\nu_{(D2O)}$-ν_1.

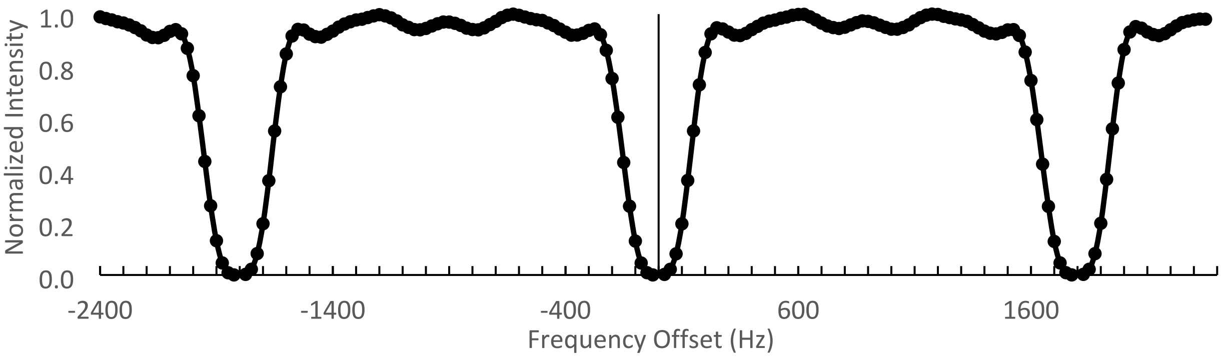

Figure S7 - Excitation profile of time-corrected JRS8 at 500 MHz using an inter-pulse of 0.556 ms (equivalent to 1800 Hz cnst10) on a D_2O sample. The offset presented at the x-axis is equal to $\nu_{(D2O)}$-ν_1.

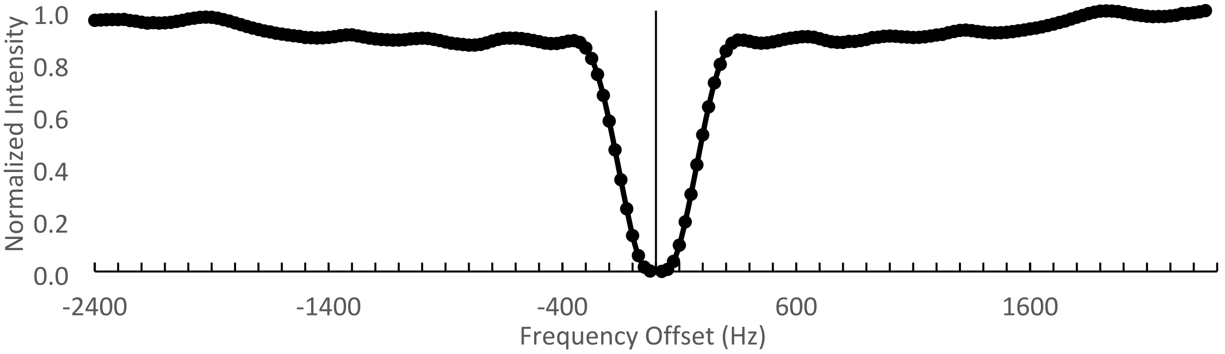

Figure S8 - Excitation profile of PE-WADE at 500 MHz on a D_2O sample. The offset presented at the x-axis is equal to $\nu_{(D2O)}$-ν_1.

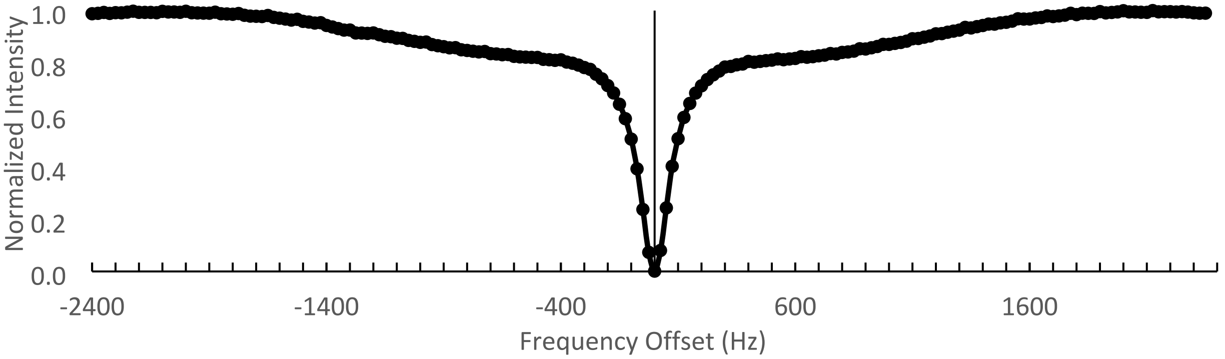

Figure S9 - Excitation profile of PURGE at 500 MHz on a D_2O sample using a 4.5 s presaturation with 50 Hz RF field strength. The offset presented at the x-axis is equal to $\nu_{(D2O)}$-ν_1.

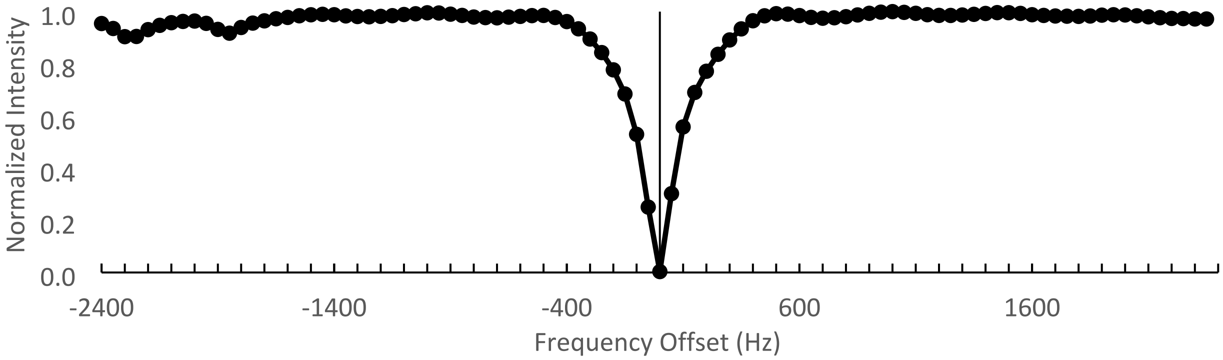

Figure S10 - Excitation profile of 1D-NOESY-pr at 500 MHz on a D_2O sample using a 4.5 s presaturation with 50 Hz RF field strength and 2 ms of mixing period. The offset presented at the x-axis is equal to $\nu_{(D2O)}$-ν_1.

Comparison of the suppression performance of Robust5, JRS8 and PE-WADE:

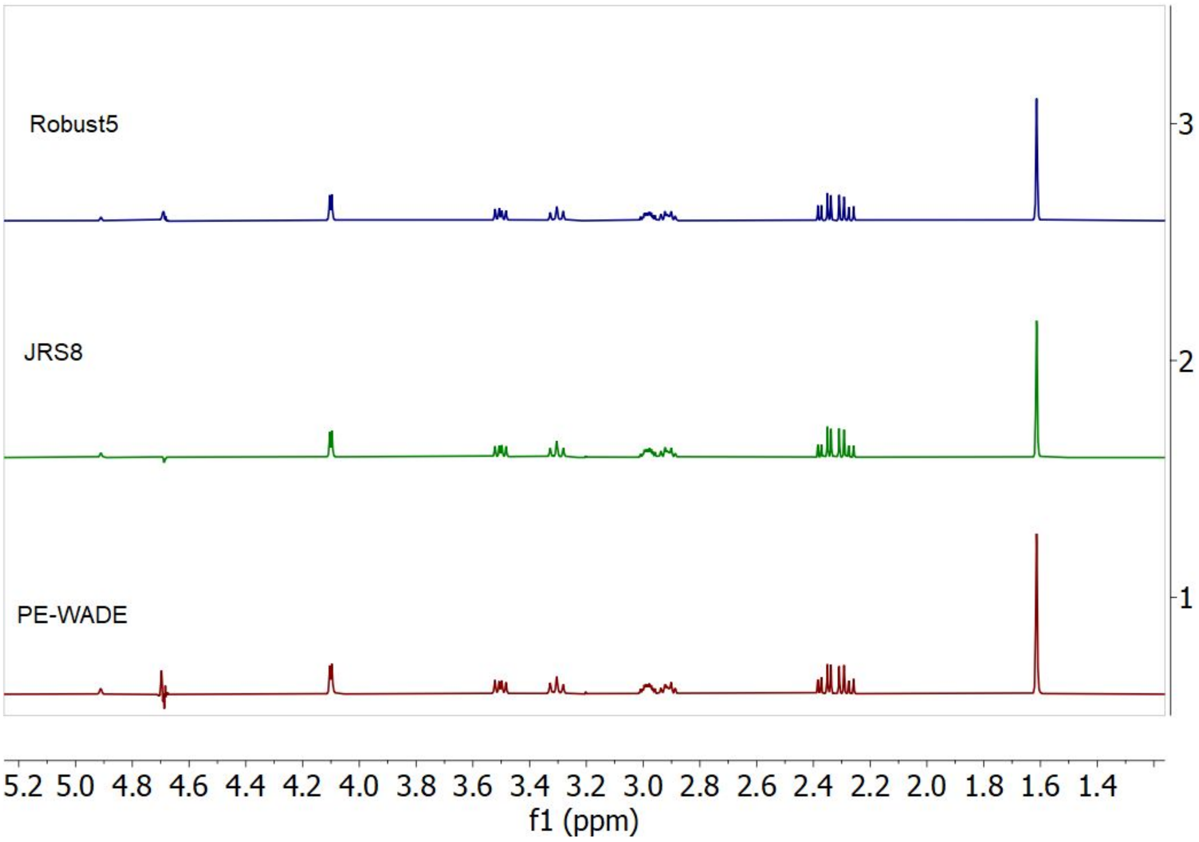

Figure S11 – Solvent suppression performance at 500 MHz of Robust5, JRS8 and PE-WADE for a no-D sample of kainic acid with maleic acid at approximately 4 mg/mL each. The intensities of the spectra were normalized using maleic acid.

Measurement Uncertainty estimation:

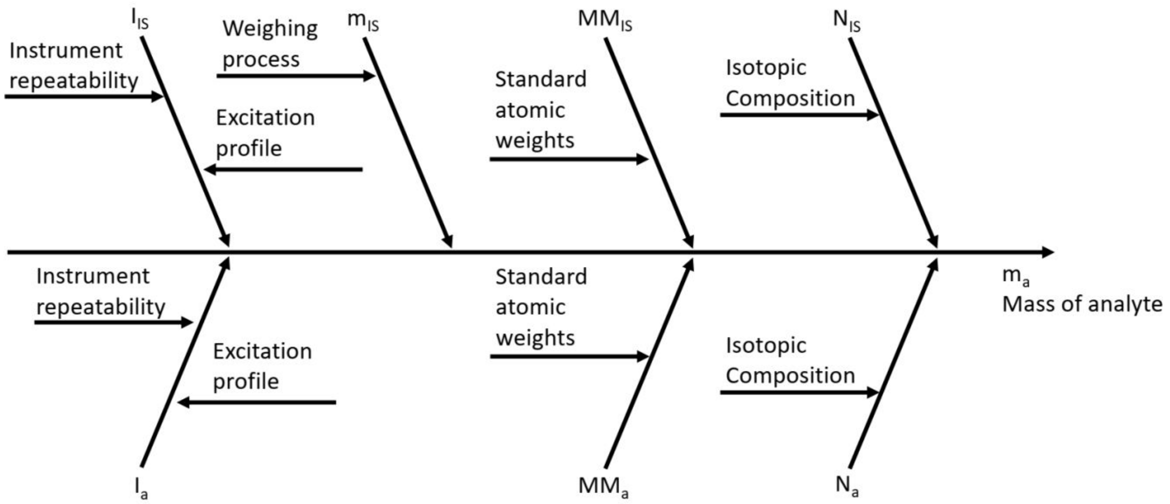

Figure S12 – Cause-effect diagram used for estimating the measurement uncertainty of qNMR measurements in the work. The excitation profile and relaxation loss input quantities are not considered for regular measurements where solvent suppression is not used.

Table S1 – Uncertainty budget for a Robust5 experiment showing the input quantities and their respective uncertainty components. The terms in brackets on the first column are the keys used for the variables in the R scripts

Input Quantity	Input u	Sensitivity Coefficient	Unit Adjusted u	u²	% u²
**Signal intensity of analyte (Ia)**	729	1.70E-05	0.01240	1.54E-04	4.0
**Signal intensity of IS (Iis)**	920	-5.00E-06	0.00460	2.11E-05	0.6
**Equivalent nuclei of analyte signal (Na)**	0.000079	-4.00E+00	0.00032	9.99E-08	0.003
**Equivalent nuclei of IS signal (Nis)**	0.000158	2.00E+00	0.00032	9.99E-08	0.003
**Molar mass of analyte (MMa)**	0.0062	1.70E-02	0.00011	1.11E-08	0.0003
**Molar mass of IS (MMis)**	0.0025	-3.50E-02	0.00009	7.69E-09	0.0002
**Mass of IS (mis)**	0.010	1.20E+00	0.01176	1.38E-04	3.6
**Purity of IS (Pis)**	0.0017	4.00E+00	0.00679	4.61E-05	1.2
**Excitation profile - analyte (exc)**	0.010	4.00E+00	0.04159	1.73E-03	45.0
**Excitation profile - analyte (exc2)**	0.010	-4.00E+00	0.04159	1.73E-03	45.0

Table S2 – Uncertainty budget for an experiment without solvent suppression showing the input quantities and their respective uncertainty components. The terms in brackets on the first column are the keys used for the variables in the R scripts

Input Quantity	Input u	Sensitivity Coefficient	Unit Adjusted u	u²	% u²
**Signal intensity of analyte (Ia)**	290	6.70E-05	0.01942	3.77E-04	33.0
**Signal intensity of IS (Iis)**	1552	-1.60E-05	0.02484	6.17E-04	54.0
**Equivalent nuclei of analyte signal (Na)**	0.000077	-4.80E+00	0.00037	1.37E-07	0.012
**Equivalent nuclei of IS signal (Nis)**	0.000154	2.40E+00	0.00037	1.37E-07	0.012
**Molar mass of analyte (MMa)**	0.0060	2.10E-02	0.00013	1.60E-08	0.0014
**Molar mass of IS (MMis)**	0.0026	-4.10E-02	0.00011	1.11E-08	0.0010
**Mass of IS (mis)**	0.010	9.40E-01	0.00944	8.91E-05	7.8
**Purity of IS (Pis)**	0.0017	4.80E+00	0.00821	6.74E-05	5.9

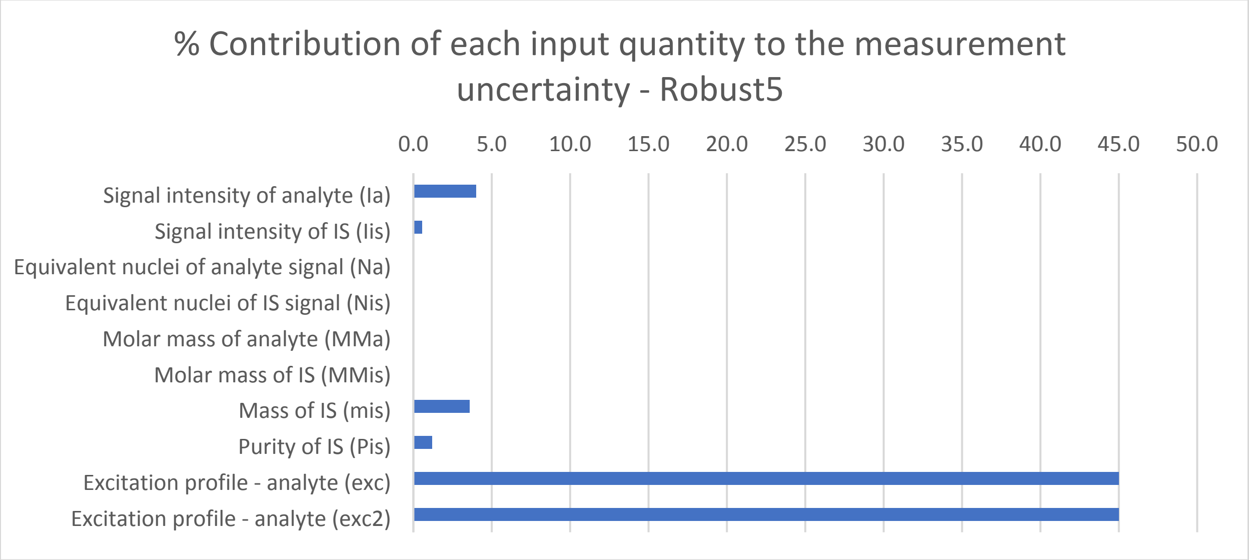

Figure S13 – Relative contributions of each uncertainty component to the overall uncertainty in Robust5. The relative contributions of some input quantities are too small to be visualized but their actual values can be seen in table S1.

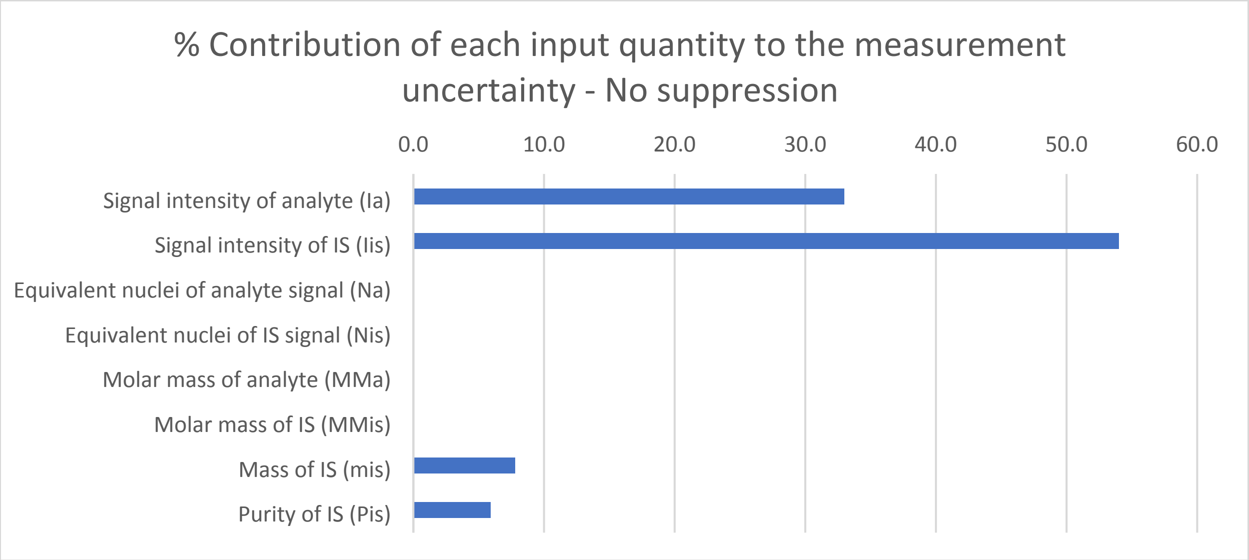

Figure S14 – Relative contributions of each uncertainty component to the overall uncertainty in an experiment without solvent suppression. The relative contributions of some input quantities are too small to be visualized but their actual values can be seen in table S2.

R script for measurement uncertainty estimation using Monte Carlo Simulation:

```
## MC simulation for uncertainty in NMR with IS and solvent suppression

m <- 100000 #Number of simulations to run for molar mass uncertainty
M <- 10000 # Number of Monte Carlo Simulations
exc <- 1.03 #The relative standard uncertainty in % associated with the excitation profile of the sequence used
Ia_raw <- c(704081.51, 680485.27, 678844.9, 678901.44) #The measured analyte area - directly from integration
Iis_raw <- c(813424.17, 815439.72, 810982.8, 812624.88) #The measured IS area - directly from integration
t <- 0.01478296 #Length of the suppression sequence
Na <- 3 #Number of equivalent nuclei of the analyte
Nis <- 2 #Number of equivalent nuclei of the IS
Formula_a <- c(10,17,1,5) #Formula of the analyte - has to be written numerically in the following order c(C,H,N,O)
Formula_is <- c(4,4,0,4) #Formula of the analyte - has to be written numerically in the following order c(C,H,N,O)
Pis <- 0.9999 #Purity of the internal standard
u_Pis <- 0.0017
mis <- 3.412 #Mass of the internal standard
u_mis <- 0.01

## Molar mass calculation and uncertainty
C <- runif(m, 12.0096, 12.0116)
H <- runif(m, 1.00784, 1.00811)
O <- runif(m, 15.99903, 15.99977)
N <- runif(m, 14.00643, 14.00728)

MMa <- Formula_a[1]*C + Formula_a[2]*H + Formula_a[3]*N + Formula_a[4]*O
MMis <- Formula_is[1]*C + Formula_is[2]*H + Formula_is[3]*N + Formula_is[4]*O

ma.x = numeric(M)
set.seed(931) #any number - starting from the same seed ensures that the same results will be obtained if the simulation is repeated.
for (i in 1:M) {
  Ia_raw_sim <- rnorm(1, mean(Ia_raw), sd(Ia_raw)/sqrt(4))
  exc_profile <- rnorm(1, 1, exc/100)
  exc_profile2 <- rnorm(1, 1, exc/100) #this component has to be drawn from the MC sample twice, otherwise it ends up being the same number and cancels out the uncertainty contribution associated with it

  Na_u <- runif(1, Na*0.99972, Na*0.99999) #Number of equivalent nuclei considering isotopic abundance of 1H - uniform distribution between 0.99972 and 0.99999 for each H atom
  Nis_u <- runif(1, Nis*0.99972, Nis*0.99999)

  MMa_sim <- runif(1, quantile(MMa, 0.025), quantile(MMa, 0.975))
  MMis_sim <- runif(1, quantile(MMis, 0.025), quantile(MMis, 0.975))

  mis_sim <- rnorm(1, mis, u_mis)
  Pis_sim <- rnorm(1, Pis, u_Pis)

  ma.x[i] =
(Ia_raw_sim*exc_profile)*Nis_u*MMa_sim*mis_sim*Pis_sim)/(Iis_raw_sim*exc_profile2*)*Na_u*MMis_sim
)
}
mean(ma.x)
sd(ma.x)

print(paste("The measured sample mass is", round(mean(ma.x),2), "mg with a standard associated uncertainty of", round(sd(ma.x),2), "mg"))

## End of the R script
```

JRS8 pulse program:

```
;JRS8tc.bg
;avance-version (09/01/2025)
;1D sequence
;water suppression using JRS8 pulse sequence with gradients
;using double echo
;Bruno Garrido, Lucas J. Carvalho, Ian Burton and Pearse McCarron, 2025
:
;JRS8 pulses from:
;Brenner et al., J. Magn. Reson. 288, 100-1008 (2018)
;
;using the time correction described in:
;Wang et al., J. Magn. Reson. 206, 205 - 209 (2010)
;
;
;$CLASS=HighRes
;$DIM=1D
;$TYPE=
;$SUBTYPE=
;$COMMENT=

#include <Avance.incl>
#include <Grad.incl>

"d19 = (1/(cnst10))"
"acqt0 = 0"

"d20=(d19)- (2*(p27*1.000 + p27*1.099)/3.1416)"
"d21=(d19)- (2*(p27*1.099 + p27*0.469)/3.1416)"
"d22=(d19)- (2*(p27*0.469 + p27*1.116)/3.1416)"
"d23=(d19)- (2*(p27*1.116 + p27*1.116)/3.1416)"
"d24=(d19)- (2*(p27*1.116 + p27*0.469)/3.1416)"
"d25=(d19)- (2*(p27*0.469 + p27*1.099)/3.1416)"
"d26=(d19)- (2*(p27*1.099 + p27*1.000)/3.1416)"

1 ze
2 30m
  d1
  10u pl1:f1
  p1 ph1
  50u UNBLKGRAD

  p16:gp1
  d16 pl18:f1
  p27*1.000 ph3
  d20
  p27*1.099 ph4
  d21
  p27*0.469 ph4
  d22
  p27*1.116 ph5
  d23
  p27*1.116 ph4
  d24
  p27*0.469 ph5
  d25
  p27*1.099 ph5
  d26
  p27*1.000 ph6
  50u
  p16:gp1
  d16

  4u

  p16:gp2
  d16
  p27*1.000 ph7
  d20
  p27*1.099 ph8
```

```
  d21
  p27*0.469 ph8
  d22
  p27*1.116 ph9
  d23
  p27*1.116 ph8
  d24
  p27*0.469 ph9
  d25
  p27*1.099 ph9
  d26
  p27*1.000 ph10
  p16:gp2
  d16

  4u BLKGRAD
  go=2 ph31
  30m mc #0 to 2 F0(zd)
exit

ph1=0 2
ph3=0 0 1 1 2 2 3 3
ph4=1 1 2 2 3 3 0 0
ph5=3 3 0 0 1 1 2 2
ph6=2 2 3 3 0 0 1 1
ph7=0
ph8=1
ph9=3
ph10=2
ph31=0 2 2 0 0 2 2 0

;pl1 : f1 channel - power level for pulse (default)
;pl18: f1 channel - power level for 3-9-19-pulse (watergate)
;p1 : f1 channel -  90 degree high power pulse
;p16: homospoil/gradient pulse
;p27: f1 channel -  90 degree pulse at pl18
;cnst10: distance to next null in Hz
;d1 : relaxation delay; 1-5 * T1
;d16: delay for homospoil/gradient recovery
;d19: delay for binomial water suppression
;     d19 = (1/(2*d)), d = distance of next null (in Hz)
;ns: 8 * n, total number of scans: NS * TD0
;ds: 4

;use gradient ratio:    gp 1 : gp 2
;                         34 :   22

;for z-only gradients:
;gpz1: 34%
;gpz2: 22%

;use gradient files:
;gpnam1: SMSQ10.100
;gpnam2: SMSQ10.100

;$Id: JRS8tc.bg,v 1.0 2025/01/09 14:11:32 ber Exp $

## End of the pulse program
```

PE-WADE pulse program:

```
;PEWADE
;Solvent supression using perfect echo and water irradiation devoid (WADE) pulses
;Bruno Garrido, Lucas J. Carvalho, Ian Burton, Pearse McCarron
;
;
;Based on
;Perfect Echo Watergate Sequence
;Ralph W. Adams, Chloe M. Holroyd, Juan A. Aguilar, Mathias Nilsson and Gareth A. Morris
;Chem. Commun., 2013,49, 358-360
;
;$CLASS=HighRes
;$DIM=1D
;$TYPE=
;$SUBTYPE=
;$COMMENT=


#include <Avance.incl>
#include <Grad.incl>

"acqt0=-4u"
"p20=p16*2"
"d19=(1/(cnst10*2))"

1 ze
2 30m
  d1
  50u UNBLKGRAD
  p20:gp3*EA
  d16
  d16
  p20:gp4*EA
  d16
  d16
        10u pl1:f1
  p1 ph1

  50u UNBLKGRAD
  p16:gp1
  d16 pl18:f1

        (p12:sp1 ph3):f1

  50u
  p16:gp1
  d16 pl1:f1

  p1 ph10


  50u
  p16:gp2
  d16 pl18:f1

        (p12:sp1 ph5):f1

  p16:gp2
  d16
  50u BLKGRAD

  go=2 ph31
  30m mc #0 to 2 F0(zd)
exit


ph1=0 2
ph3=0 0 1 1 2 2 3 3

ph5=0 0 0 0 0 0 0 0 1 1 1 1 1 1 1 1
```

```
    2 2 2 2 2 2 2 2 3 3 3 3 3 3 3 3

ph10=1
ph31=0 2 2 0 0 2 2 0 2 0 0 2 2 0 0 2


;pl1 : f1 channel - power level for pulse (default)
;pl18: f1 channel - power level for W5-pulse (watergate)
;sp1 :  f1 channel - shaped pulse 180 degree
;p1 : f1 channel -  90 degree high power pulse
;p12 : shaped WADE pulse lenght
;p16: homospoil/gradient pulse
;p27: f1 channel -  90 degree pulse at pl18
;d1 : relaxation delay; 1-5 * T1
;d16: delay for homospoil/gradient recovery
;d19: delay for binomial water suppression
;     d19 = (1/(2*d)), d = distance of next null (in Hz)
;cnst10 = distance to next null (in Hz)
;NS: 8 * n, total number of scans: NS * TD0
;DS: 4


;use gradient ratio:    gp 1 : gp 2 :gp 3: gp 4
;                         34 :   22 : -34 : -22

;for z-only gradients:
;gpz1: 34%
;gpz2: 22%
;gpz3: -34%
;gpz4: -22%

;use gradient files:
;gpnam1: SINE.100
;gpnam2: SINE.100
;gpnam3: SINE.100
;gpnam4: SINE.100

;Use shaped pulse wadepix_NP_2_rf0.00_34.595p1_bw0.82B1.mrf - download from
https://github.com/manuvsub/WADE_NOESY
;Adjust bandwidth according to instructions on the pulse readme file using the "pulse" AU in
topspin to calculate RF power


;$Id: pewade,v 1.0 2025/01/09 14:05:08 ber Exp $

## End of the pulse program
```

T1 measurement pulse program:

```
;t1ir-pew5.bg
;avance-version (09/01/2025)
;T1 measurement using inversion recovery
;with solvent suppression using double gradient echo and perfect echo W5
;Bruno Garrido, Lucas J. Carvalho, Ian Burton and Pearse McCarron, 2025
;Perfect echo W5 described in:
;
;Ralph W. Adams, Chloe M. Holroyd, Juan A. Aguilar, Mathias Nilsson and Gareth A. Morris
;Chem. Commun., 2013,49, 358-360
;
;$CLASS=HighRes
;$DIM=2D
;$TYPE=
;$SUBTYPE=
;$COMMENT=

#include <Avance.incl>
#include <Grad.incl>

"p2=p1*2"
"d11=30m"

"acqt0=-p1*2/3.1416"

1 ze
2 d1
  p2 ph1
  vd
  p1 ph1

  50u UNBLKGRAD
  p16:gp1
  d16 pl18:f1
  p27*0.087 ph3
  d19*2
  p27*0.206 ph3
  d19*2
  p27*0.413 ph3
  d19*2
  p27*0.778 ph3
  d19*2
  p27*1.491 ph3
  d19*2
  p27*1.491 ph4
  d19*2
  p27*0.778 ph4
  d19*2
  p27*0.413 ph4
  d19*2
  p27*0.206 ph4
  d19*2
  p27*0.087 ph4
  50u
  p16:gp1
  d16 pl1:f1

  p1 ph10

  50u
  p16:gp2
  d16 pl18:f1
  p27*0.087 ph5
  d19*2
  p27*0.206 ph5
  d19*2
  p27*0.413 ph5
  d19*2
  p27*0.778 ph5
  d19*2
```

```
  p27*1.491 ph5
  d19*2
  p27*1.491 ph6
  d19*2
  p27*0.778 ph6
  d19*2
  p27*0.413 ph6
  d19*2
  p27*0.206 ph6
  d19*2
  p27*0.087 ph6
  p16:gp2
  d16
  50u BLKGRAD

  go=2 ph31
  d11 wr #0 if #0 ivd
  lo to 1 times td1
exit

ph1=0 2
ph3=0 0 1 1 2 2 3 3
ph4=2 2 3 3 0 0 1 1
ph5=0 0 0 0 0 0 0 0 1 1 1 1 1 1 1 1
    2 2 2 2 2 2 2 2 3 3 3 3 3 3 3 3
ph6=2 2 2 2 2 2 2 2 3 3 3 3 3 3 3 3
    0 0 0 0 0 0 0 0 1 1 1 1 1 1 1 1
ph10=1
ph31=0 0 2 2 1 1 3 3

;pl1 : f1 channel - power level for pulse (default)
;pl18: f1 channel - power level for 3-9-19-pulse (watergate)
;p1 : f1 channel -  90 degree high power pulse
;p2 : f1 channel - 180 degree high power pulse
;p16: homospoil/gradient pulse
;p27: f1 channel -  90 degree pulse at pl18
;d1 : relaxation delay; 1-5 * T1
;d11: delay for disk I/O                            [30 msec]
;d16: delay for homospoil/gradient recovery
;d19: delay for binomial water suppression
;     d19 = (1/(2*d)), d = distance of next null (in Hz)
;vd : variable delay, taken from vd-list
;ns: 8 * n
;ds: 4
;td1: number of experiments = number of delays in vd-list
;FnMODE: undefined

;use gradient ratio:    gp 1 : gp 2
;                         34 :   22

;for z-only gradients:
;gpz1: 34%
;gpz2: 22%

;use gradient files:
;gpnam1: SINE.100
;gpnam2: SINE.100

;define VDLIST

;this pulse program produces a ser-file (PARMOD = 2D)

;$Id: t1ir,v 1.13 2012/01/31 17:49:28 ber Exp $

## End of the pulse program
```
